# Smartphone Ownership, Smartphone Utilization, and Interest in Using Mental Health Apps to Address Substance Use Disorders: Literature Review and Cross-sectional Survey Study Across Two Sites

**DOI:** 10.2196/38684

**Published:** 2022-07-07

**Authors:** Michael Hsu, Bianca Martin, Saeed Ahmed, John Torous, Joji Suzuki

**Affiliations:** 1 Department of Psychiatry Brigham and Women's Hospital Boston, MA United States; 2 West Ridge Center Rutland Regional Medical Center Rutland, VT United States; 3 Digital Psychiatry Division Department of Psychiatry Beth Israel Deaconess Medical Center Boston, MA United States

**Keywords:** smartphone, mobile phone, addiction, substance use, phone ownership, health equity, digital psychiatry, digital phenotyping, phone applications, substance abuse, mHealth, phone utilization, mental health, mindfulness, digital mental health

## Abstract

**Background:**

In recent years, there has been increasing interest in implementing digital technologies to diagnose, monitor, and intervene in substance use disorders. Smartphones are now a vehicle for facilitating telepsychiatry visits, measuring health metrics, and communicating with health care professionals. In light of the COVID-19 pandemic and the movement toward web-based and hybrid clinic visits and meetings, it has become especially salient to assess phone ownership among individuals with substance use disorders and their comfort in navigating phone functionality and using phones for mental health purposes.

**Objective:**

The aims of this study were to summarize the current literature around smartphone ownership, smartphone utilization, and the acceptability of using smartphones for mental health purposes and assess these variables across two disparate substance use treatment sites.

**Methods:**

We performed a focused literature review via a search of two academic databases (PubMed and Google Scholar) for publications since 2007 on the topics of smartphone ownership, smartphone utilization, and the acceptability of using mobile apps for mental health purposes among the substance use population. Additionally, we conducted a cross-sectional survey study that included 51 participants across two sites in New England—an inpatient detoxification unit that predominantly treats patients with alcohol use disorder and an outpatient methadone maintenance treatment clinic.

**Results:**

Prior studies indicated that mobile phone ownership among the substance use population between 2013 and 2019 ranged from 83% to 94%, while smartphone ownership ranged from 57% to 94%. The results from our study across the two sites indicated 96% (49/51) mobile phone ownership and 92% (47/51) smartphone ownership among the substance use population. Although most (43/49, 88%) patients across both sites reported currently using apps on their phone, a minority (19/48, 40%) reported previously using any apps for mental health purposes. More than half of the participants reported feeling at least neutrally comfortable with a mental health app gathering information regarding appointment reminders (32/48, 67%), medication reminders (33/48, 69%), and symptom surveys (26/45, 58%). Most patients were concerned about privacy (34/51, 67%) and felt uncomfortable with an app gathering location (29/47, 62%) and social (27/47, 57%) information for health care purposes.

**Conclusions:**

The majority of respondents reported owning a mobile phone (49/51, 96%) and smartphone (47/51, 92%), consistent with prior studies. Many respondents felt comfortable with mental health apps gathering most forms of personal information and with communicating with their clinician about their mental health. The differential results from the two sites, namely greater concerns about the cost of mental health apps among the methadone maintenance treatment cohort and less experience with downloading apps among the older inpatient detoxification cohort, may indicate that clinicians should tailor technological interventions based on local demographics and practice sites and that there is likely not a one-size-fits-all digital psychiatry solution.

## Introduction

Handheld phones evolved at a lightning pace over the past 2 decades. Once devices that were solely used to text or call, smartphones now connect millions through social media, track health metrics, and have GPS-sensing capabilities. Smartphones also have a burgeoning role in telepsychiatry, which has been widely adopted during the COVID-19 pandemic. Tens of thousands of mental health apps are available through app stores. Digital phenotyping, which involves using passively and continuously collected sensory and user data to track movement, phone utilization, and communication, has potential apps for relapse prediction in schizophrenia [1], relapse prediction in bipolar disorder [2], and depressive episode detection [3].

Smartphone apps have expanded and can be used for the diagnosis, monitoring, and treatment of substance use disorders [4]. For instance, Reset-O is the first US Food and Drug Administration–approved prescription mobile app for the treatment of opioid use disorder with evidence of improving abstinence and treatment retention [5]. Additionally, a smartphone app may be able to detect an opioid overdose by using a short-range active sonar [6]. Further, A-CHESS (Addiction-Comprehensive Health Enhancement Support System) is a mobile app suite that buttresses alcohol recovery through features such as delivering alerts to patients when they approach known triggers, such as a bar or liquor store, via GPS [7].

With smartphones playing an increasingly integral role in the delivery of substance use care, addressing equity and understanding the adoption and utilization of smartphones and the acceptability of related technologies have become ever more pressing. We performed a nonsystematic review of literature around smartphone ownership and utilization among individuals with substance use disorders, and we present original data from our 2-site, cross-sectional survey study assessing smartphone ownership, smartphone utilization, and the acceptability of using mental health apps. We hypothesize that a vast majority of individuals own smartphones and would be open to using smartphone apps to address substance use. The aim of this study is to inform clinicians who plan to develop or implement digital interventions for patients with substance use disorders on how to anticipate and optimize adoption and engagement.

## Methods

### Literature Review

The purpose of the literature review was to identify articles that assessed smartphone or phone ownership and/or utilization to answer the following questions: what proportion of patients with substance use disorders own mobile phones or smartphones, how did they utilize their phones, and how open were they to mobile health (mHealth) intervention through smartphones?

A nonsystematic search was conducted in January 2022 within PubMed and Google Scholar, using the key terms *smartphone ownership*, *phone ownership*, *access to a mobile phone*, *access to a smartphone*, *substance use disorder*, *addiction*, and *substance abuse*, by two independent researchers. The selected publication types included primary survey or questionnaire studies, systematic or nonsystematic reviews, and secondary research studies based on prior survey or census data published in or after 2007 (the year that the Apple iPhone was introduced). Other inclusion criteria included articles written in the English language and participants with substance use disorders. The exclusion criteria included clinical trials of specific digital interventions, such as a smartphone app, as the purpose of this study was to understand general smartphone ownership and use. Other exclusion criteria included papers written in foreign languages and studies on participants with behavioral addictions, such as internet gaming disorder, internet addiction, or gambling disorder, as opposed to substance use disorders. A total of 8 abstracts were identified by using these criteria. Study purposes, study designs, descriptions of methods, sample sizes, sample demographics, smartphone or phone ownership rates, and smartphone utilization and acceptability data were extracted.

### Cross-sectional, 2-Site Survey Study

We developed a survey that closely mirrored the one developed by Torous et al [8] (Textbox 1). The 5- to 10-minute survey was completed via paper and pencil and included sections on demographics, phone ownership, phone utilization, and the acceptability of using phones for mental health care purposes. Surveys were administered between June 2021 and February 2022 at two sites—a level 4 inpatient detoxification unit at a community hospital in Massachusetts (Brigham and Women’s Faulkner Hospital [BWFH]) that predominantly treats individuals with alcohol use disorder and an outpatient methadone clinic in Rutland, Vermont (West Ridge Clinic). Participants were offered a US $5 *Dunkin’ Donuts* gift card for completing this study. There was a total of 51 participants from both sites—22 from the inpatient detoxification unit and 29 from the outpatient methadone clinic. Descriptive statistics were used to summarize the data; all analyses were performed by using Stata Statistical Software: Release 17 (StataCorp LLC). Demographic and mobile phone use variables for those at the detoxification unit and those at the outpatient methadone clinic were compared by using a chi-square test for categorical variables and a 2-tailed Student *t* test for continuous variables.

**Survey questions. Demographic questions are excluded.**

**Questions**

1. Do you currently own any type of phone?
  If no, for what reason do you not have a phone?
2. Is your phone a smartphone (eg, iPhone, Android, etc)?
3. What is the name of your smartphone (eg, Samsung Galaxy)?
4. How comfortable are you sending text messages on your phone?
5. What type of payment plan do you use for text messages?
6. Do you download apps onto your phone?
7. Have you ever downloaded an app for your mental health?
8. Do you currently use any apps for your phone?
9. How comfortable or uncomfortable would you feel about a mental health app gathering and/or sending the following data from your smartphone to your clinician in the context of your care?
  Appointment reminders
  Medication reminders
  Symptom surveys (eg, survey questions about your mood or thoughts throughout the day)
  Your location (phone GPS sensor)
  Your social information (call and text logs without any phone numbers or context of messages; eg, how many people you called and for how long)
  Coaching for healthy living (eg, exercise, sleep, and diet)
  Mindfulness or therapy exercises
  Communicating with my clinician about my mental health
10. Select up to 3 top concerns you may have about mental health apps or apps for substance use disorders:
  Privacy
  Accuracy of recommendations from app
  Hard to use
  Sharing information with clinician
  Cost
  Time
  Hard to set up
11. Select up to 3 top benefits you may see in mental health apps or apps for substance use disorders:
  Privacy
  Accuracy of recommendations from app
  Easy to use
  Sharing information with clinician
  Cost
  Time
  Easy to set up

### Ethical Considerations

This study was approved and monitored by the Mass General Brigham Institutional Review Board (approval number 2020P001656) and Rutland Regional Hospital Institutional Review Board (approval number 2020P001656/RRMC24). This study was identified as human subjects research and was classified as exempt by the Mass General Brigham Institutional Review Board, given the minimal risk to subjects and the use of a survey tool with no identifiable information obtained. This study adheres to the ethical guidelines set forth by the Mass General Brigham Human Research Protection Program [9]. Participants were consented prior to the survey study and were given the choice to opt out of specific questions or this study entirely at any time. No personal health or identifiable information was recorded.

## Results

### Participant Characteristics

For the inpatient detoxification cohort, the study team approached 35 participants, of whom 25 consented to answering the survey and 22 completed it (response rate: 22/35, 63%). Further, 2 participants were discharged from detoxification prior to completion, and 1 participant dropped out.

The BWFH inpatient detoxification sample skewed toward male (12/22, 55%) and White (20/22, 91%) patients, and the West Ridge Clinic sample skewed toward female (18/29, 62%) and White (23/29, 79%) patients (Table 1). Notably, the West Ridge Clinic had a relatively higher proportion of individuals experiencing homelessness (10/28, 36%) compared to that of the inpatient detoxification clinic (1/22, 5%), though this did not reach statistical significance (*P*=.10). There were no additional differences in demographics between the two clinic sites except for the fact that the mean age at the methadone maintenance treatment (MMT) clinic was significantly lower (48.71 vs 36.04 years; *P*<.001).

**Table 1:** Summary of participant demographic characteristics (N=51).

Variable		All participants (N=51)	BWFH^a^ inpatient detoxification clinic (n=22)	West Ridge Clinic (n=29)	*P* value	
Age (years), mean (SD)		41.47 (13.0)	48.71 (11.8)	36.04 (11.2)	.001	
**Sex, n (%)**						.24
	Male	23 (45)	12 (55)	11 (38)		
	Female	28 (55)	10 (45)	18 (62)		
**Race/ethnicity, n (%)**						.32
	Black or African American	1 (2)	0 (0)	1 (4)		
	White	43 (84)	20 (91)	23 (79)		
	Hispanic or Latinx	3 (6)	2 (9)	1 (4)		
	Other	3 (6)	0 (0)	3 (10)		
	Alaska Native	1 (2)	0 (0)	1 (4)		
**Education (highest level completed), n (%)**						.20
	Completed high school or General Educational Development	13 (25)	5 (23)	8 (28)		
	Some high school	7 (14)	1 (4)	6 (21)		
	Completed college or associate degree	6 (12)	3 (14)	3 (10)		
	Some college or associate degree	17 (33)	7 (32)	10 (34)		
	Graduate school	8 (16)	6 (27)	2 (7)		
**Work, n (%)**						.18
	Full-time employment	12 (23)	4 (18)	8 (28)		
	Part-time employment	5 (10)	1 (5)	4 (14)		
	Unemployed	18 (35)	7 (32)	11 (38)		
	SSD^b^ or SSI^c^	9 (18)	4 (18)	5 (17)		
	Other	3 (6)	2 (9)	1 (3)		
	Retired	4 (8)	4 (18)	0 (0)		
**Residence (n=50)^d^, n (%)**						.10
	Own or rent apartment	18 (36)	10 (45)	8 (29)		
	Family or friends	10 (20)	6 (27)	4 (14)		
	Single room occupancy	8 (16)	4 (18)	4 (14)		
	Halfway house	3 (6)	1 (5)	2 (7)		
	Homeless	11 (22)	1 (5)	10 (36)		

^a^BWFH: Brigham and Women’s Faulkner Hospital.

^b^SSD: Social Security Disability.

^c^SSI: Supplemental Security Income.

^d^One participant did not answer the question regarding place of residence.

### Phone Ownership in the Substance Use Population

Prior studies indicated that mobile phone ownership among the substance use population from 2013 to 2019 ranged from 83% to 94%, while smartphone ownership ranged from 57% to 94% [10-17] (Table 2). Individuals of the baby boomer generation (aged >52 years) may be less likely to own a mobile phone with app capability when compared to Generation X (age: range 36 to 51 years; *P*=.001; odds ratio 3.52, 95% CI 1.65-7.52) and millennials (age: range 18 to 35; *P*<.001; odds ratio 4.53, 95% CI 2.19-9.35) [14].

In our study, a large proportion of respondents reported owning a mobile phone (49/51, 96%) and smartphone (47/51, 92%). All patients (22/22, 100%) at the inpatient detoxification clinic reported owning a mobile phone, while 27 of 29 patients (93%) reported owning a mobile phone at the outpatient methadone clinic. Of the 49 respondents who owned mobile phones across both sites, 47 (96%) categorized them as smartphones; 2 participants opted out of this question.

**Table 2:** Mobile phone and smartphone ownership among individuals with substance use disorders across studies.

Authors, year	Patient population	Sample size, N	Mobile phone ownership, %	Smartphone ownership, %
McClure et al, 2013 [16]	Adult patients who were undergoing substance abuse treatment and were enrolled at 8 drug-free psychosocial or opioid-replacement therapy clinics in Baltimore	266	91	N/A^a^
Dahne and Lejuez, 2015 [12]	Adult patients admitted to a residential substance use treatment center in Washington, District of Columbia	251	86.9	68.5
Tofighi et al, 2015 [17]	Adult patients with opiate dependence in an urban, safety-net office–based buprenorphine program in New York City	71	93	63
Milward et al, 2015 [13]	Patients enrolled in 4 UK community drug treatment services (74% were undergoing treatment for heroin addiction)	398	83	57
Masson et al, 2018 [11]	Adult patients enrolled in methadone maintenance treatment in San Francisco	178	87	N/A
Ashford et al, 2018 [14]	Adult patients in 4 intensive outpatient substance use disorder treatment facilities in Philadelphia	259	93.8	64.1
Curtis et al, 2019 [10]	Adolescents (aged 13-17 years) and emerging adults (aged 18-35 years) engaged in outpatient substance use treatment in the Southwest and Northeast regions of the United States	164	92.2	80.9
Tofighi et al, 2019 [15]	Adult patients enrolled in an inpatient detoxification program at a safety-net tertiary referral center in New York City	206	86	66

^a^N/A: not applicable.

### Phone Utilization in the Substance Use Population

Based on our review of the literature, 79% to 96% of individuals with substance use disorders have phones with text messaging capabilities [12,15,16], with one study published in 2015 suggesting that 55% of mobile phone owners use their phones to text daily [13]. Between 61% to 68% of individuals use their mobile phones to access the internet [10,12,15], and 61.3% of adult smartphone owners use mobile apps on their phones [12]. One study found that of individuals who accessed the internet, 80% accessed it primarily through their mobile phones, with no generational differences in terms of using phones to access the internet versus other means [14]. Masson et al [11] concluded that 40% (n=70) of their participants used their mobile devices as a reminder to take medications, while 8% (n=13) of smartphone users utilized a medication reminder app. McClure et al [16] found that 44% of their adult participants reported being called by substance use clinic staff, while 0.4% reported being texted by them.

Patients were also asked about their mobile phone use patterns in our study (Table 3). Nearly all patients across both sites (45/49, 92%) reported feeling extremely, very, or somewhat comfortable with sending text messages. Of the 47 patients who reported their type of text message payment plan, most (42/47, 89%) reported paying a flat fee for unlimited text messages. The remaining 5 patients reported either paying a flat fee for a limited text message plan (2/47, 4%) or using a pay-per-text plan (3/47, 6%). The majority of respondents (43/49, 88%) reported having downloaded apps, although this differed significantly across sites (West Ridge Clinic patients: 26/27, 96%; inpatient detoxification clinic patients: 17/22, 77%; *P*=.04). Although most patients (43/49, 88%) across both sites reported currently using apps on their phone, only a minority (19/48, 40%) reported having previously used any app for their mental health; this did not differ significantly across sites (*P*=.21).

**Table 3:** Mobile phone use patterns (N=51).

Variable			All participants (N=51), n (%)		BWFH^a^ inpatient detoxification clinic (n=22), n (%)		West Ridge Clinic (n=29), n (%)		*P* value	
**Mobile phone ownership**			49 (96)		22 (100)		27 (93)		.21	
	Smartphone (n=47)^b^	47 (100)		20 (100)		27 (100)				
**Sending text messages (n=49)^c^**										
	Extremely, very, or somewhat comfortable	45 (92)		19 (86)		26 (96)		.21		
**Text message payment plan (n=47)^d^**										.30
	Flat fee for unlimited text messages	42 (89)		19 (95)		23 (85)				
	Flat fee for limited text messages	2 (4)		1 (5)		1 (4)				
	Pay-per-text plan	3 (7)		0 (0)		3 (11)				
**Downloads apps onto phone (n=49)^e^, n (%)**			43 (88)		17 (77)		26 (96)		.04	
	Has downloaded app for mental health	19 (40)		10 (48)		9 (33)		.32		
	Currently uses any apps on phone	43 (88)		18 (82)		25 (93)		.25		
**Very, somewhat, or neutrally comfortable with mental health app gathering information (n=48)^f^**										
	Appointment reminders	32 (67)		11 (52)		21 (78)		.06		
	Medication reminders	33 (69)		12 (57)		21 (78)		.13		
	Symptom surveys	26 (58)		11 (52)		15 (63)		.49		
	Location	18 (38)		7 (33)		11 (42)		.53		
	Social information	20 (43)		8 (38)		12 (46)		.58		
	Coaching for healthy living	27 (56)		11 (52)		16 (59)		.63		
	Mindfulness or therapy exercises	31 (65)		12 (57)		19 (70)		.34		
	Communicating with clinician about mental health	30 (63)		11 (52)		19 (70)		.20		
**Perceived concerns about mental health apps**										
	Privacy	34 (67)		14 (64)		20 (69)		.69		
	Accuracy of recommendations	12 (24)		4 (18)		8 (28)		.43		
	Hard to use	9 (18)		5 (23)		4 (14)		.41		
	Sharing information with clinician	14 (28)		7 (32)		7 (24)		.54		
	Cost	15 (29)		2 (9)		13 (45)		.006		
	Time	16 (31)		5 (23)		11 (38)		.25		
	Hard to set up	11 (22)		7 (32)		4 (14)		.12		
**Perceived benefits of mental health apps**										
	Privacy	11 (22)		4 (18)		7 (24)		.61		
	Accuracy of recommendations	14 (28)		3 (14)		11 (38)		.05		
	Easy to use	22 (43)		10 (46)		12 (41)		.77		
	Sharing information with clinician	19 (37)		8 (36)		11 (38)		.91		
	Cost	8 (16)		2 (9)		6 (21)		.26		
	Time	20 (39)		8 (36)		12 (41)		.72		
	Easy to set up	18 (35)		4 (18)		14 (48)		.03		

^a^BWFH: Brigham and Women’s Faulkner Hospital.

^b^Two participants who reported owning a mobile phone did not provide information about whether it was a smartphone.

^c^Two participants did not answer questions regarding their comfort with sending text messages.

^d^Four participants did not answer questions regarding their current text message payment plan.

^e^Two participants did not answer questions regarding downloading apps onto their phones.

^f^Three participants did not answer questions regarding their comfort with mental health apps gathering personal information.

### Acceptability of Using Smartphones for Mental Health Purposes

Based on our review of the literature, 70.4% of adult participants in one study stated that they would use a relapse prevention app [14], though a different study by Curtis et al [10] suggested that 36.9% of millennials and 45.3% of the Generation Z population thought that a mobile phone app would be helpful toward recovery. In another study, 46% of participants found it unacceptable to use geolocation in a smartphone app for health care purposes [13], but 86% of surveyed adults were willing to be contacted via mobile phone by their clinicians, with telephone calls (53%) being the most preferred method when compared to text messages (41%) and letters (41%) [13]. The most preferred frequency of contact was 1 to 2 messages weekly (46%) [13]. Further, 72% of adults surveyed by Ashford et al [14] in 2018 reported that it was acceptable to receive text messages for relapse prevention. Curtis et al [10] found that 28.8% of millennials and 45.3% of the Generation Z population thought that texting could be helpful toward recovery.

**Figure 1:**
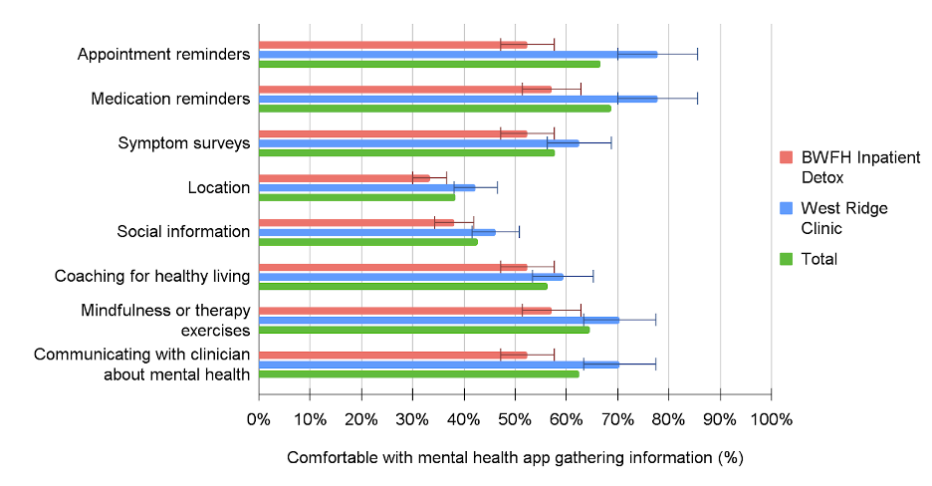
Patients' comfort with a mental health app gathering information on smartphone by clinic location. BWFH: Brigham and Women’s Faulkner Hospital.

**Figure 2:**
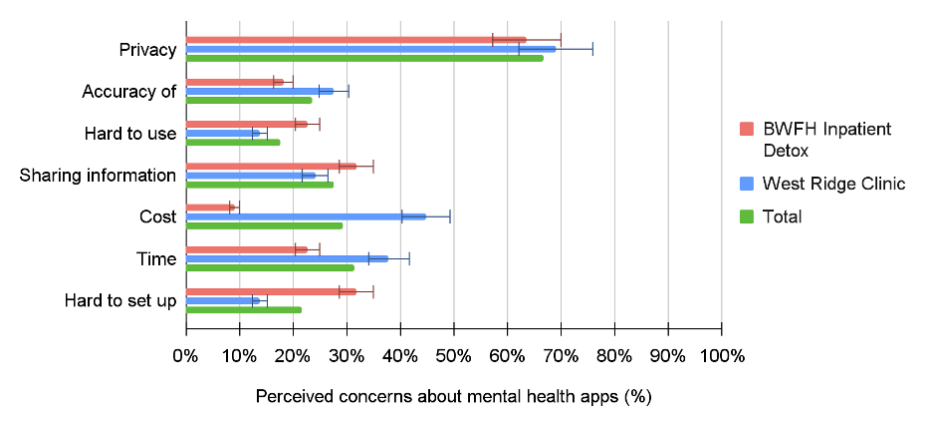
Patients' perceived concerns about mental health apps by clinic location. BWFH: Brigham and Women’s Faulkner Hospital.

**Figure 3:**
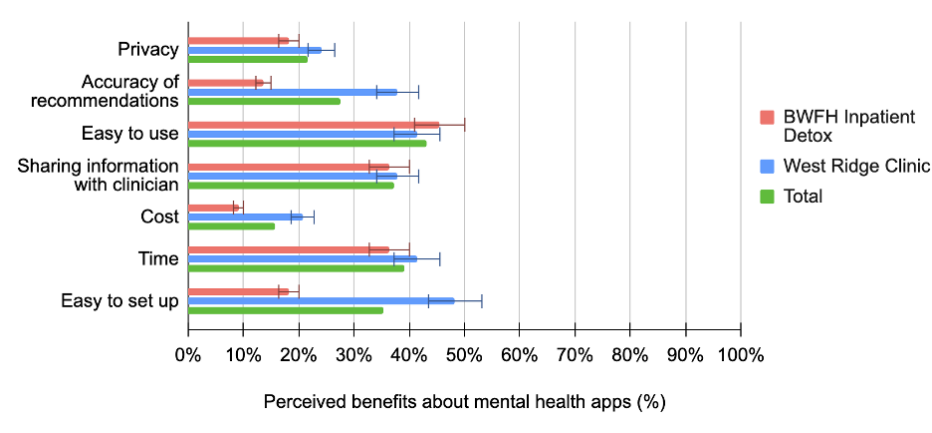
Patients' perceived benefits about mental health apps by clinic location. BWFH: Brigham and Women’s Faulkner Hospital.

Over half of all participants at both sites were at least neutrally comfortable with a mental health app gathering information regarding appointment reminders (32/48, 67%), medication reminders (33/48, 69%), and symptom surveys (26/45, 58%). Most participants also found it acceptable to use mental health apps to engage in coaching for healthy living (27/48, 56%), mindfulness or therapy exercises (31/48, 65%), and communication with their clinician about their mental health (30/48, 62%). Notably, most of our sample expressed concerns about privacy (34/51, 67%) and reported being uncomfortable with an app gathering information about location (29/47, 62%) and social information (27/47, 57%) for health care purposes. The top three noted concerns about using mental health apps were privacy, cost, and time; patients at the outpatient methadone clinic were significantly more likely to perceive cost as a top-three concern (13/29, 45% vs 2/22, 9%; *P*=.006). Overall, the top three perceived benefits of using mental health apps or apps for substance use disorders were ease of use, the ability to share information with clinicians, and time. Patients at the outpatient methadone clinic were also more likely to perceive ease of setup as a benefit of using such apps (14/29, 48% vs 4/22, 18%; *P*=.03). The acceptability results are summarized in Table 3 and Figures 1-3.

## Discussion

### Principal Findings

The overall rate of mobile phone ownership was 96% (49/51), and the overall rate of smartphone ownership was 92% (47/51). The participants recruited at the community inpatient detoxification site were overall older (mean 48.71 vs mean 36.04 years; *P*<.001), and a greater percentage were housed. Participants at the MMT clinic were more likely to report downloading apps when compared to the detoxification sample (26/27, 96% vs 17/22, 77%; *P*=.04), which could be potentially explained by the younger mean age of the methadone cohort (36.04 vs 48.71 years; *P*<.001) [18]. Most patients (43/49, 88%) reported regularly downloading and using apps on their phone, although only 40% (19/48) reported ever downloading an app specifically for mental health or substance use disorder purposes.

A majority of participants across both clinic sites indicated feeling comfortable with mental health apps gathering most forms of personal information, specifically appointment reminders (32/48, 67%), medication reminders (33/48, 69%), symptom surveys (26/45, 58%), coaching for healthy living (27/48, 56%), mindfulness or therapy exercises (31/48, 65%), and communications with their clinician about their mental health (30/48, 62%). Most individuals were uncomfortable with a mental health app tracking location (29/47, 62%) or social information (ie, their call and text logs; 27/47, 57%). The differential views on cost as a barrier to using a mental health app across the two sites (methadone clinic: 13/29, 45%; inpatient detoxification clinic: 2/22, 9%; *P*=.006) might reflect socioeconomic differences across the cohorts. As previously mentioned, the MMT sample had a relatively higher proportion of individuals experiencing homelessness (10/28, 36%) compared to that of the inpatient detoxification sample (1/22, 5%), though the comparative rates of homelessness across the two samples did not reach statistical significance (*P*=.10). Most participants cited privacy (34/51, 67%) as a primary concern with using mental health apps and reported that they would not be comfortable with geolocation (29/47, 62%) or social information tracking (27/47, 57%). The participants recruited at the MMT site were significantly more likely to perceive the ease of setting up a mobile app as a perceived benefit when compared to those of the inpatient detoxification unit (14/29, 48% vs 4/22, 18%; *P=*.03), which may again speak to participants at the MMT clinic being more comfortable with using app functionalities.

Overall, our cross-sectional study suggests that individuals with substance use disorders are generally amenable to using a smartphone app for mental health monitoring or treatment purposes. Interestingly, while smartphone ownership was slightly lower among participants in the MMT site compared to that among the detoxification site participants, which is unsurprising given that the participants at the MMT site were of lower socioeconomic status, our data suggest that the individuals recruited at the MMT site had higher digital literacy, as reflected by their comfort with downloading apps and their perception that ease of use is a benefit of using a mental health app for substance use interventions. In conclusion, clinicians should consider patient demographics, digital literacy, and practice sites when implementing mHealth interventions for substance use disorders in an equitable fashion.

### Comparison to Prior Work

To our knowledge, this study represents the first literature review of smartphone ownership, smartphone utilization, and the acceptability of using mHealth among individuals with substance use disorders and the first cross-sectional survey study to address this topic since the beginning of the COVID-19 pandemic. Smartphone and mobile phone ownership rates in our cross-sectional survey study were higher than those reported in all prior studies, likely reflecting the growing adoption of smartphones. Overall rates of downloading apps across both survey sites (43/49, 88%) were also higher than the 61% to 64% of participants who reported downloading mobile apps in a study by Dahne and Lejuez [12], which recruited patients from 2014 to 2015. The proportion of participants who felt uncomfortable with location being tracked (29/47, 62%) was slightly higher than that reported by Milward et al [13]. Comfort with utilizing specific functions of apps for substance use disorders, such as appointment reminders or social functions, and specific perceived benefits of using mobile apps for substance use disorders were not assessed in prior studies of individuals with substance use disorders.

### Strengths and Limitations

This study has several limitations. First, we performed a brief, nonsystematic review, and it is likely that relevant papers may not have been included. We attempted to strengthen the robustness of this focused literature review by utilizing two independent reviewers, two separate search engines, and broad key words to capture and screen more abstracts. Future works should incorporate a PRISMA (Preferred Reporting Items for Systematic Reviews and Meta-Analyses)-based systematic review to capture a broader range of studies. Second, the relatively small sample of our cross-sectional survey study precluded our ability to explore the impacts that race, socioeconomic status, age, and other factors have on smartphone ownership, utilization, and acceptability. Third, the predominantly White sample, especially the predominantly White inpatient detoxification cohort, limits the generalizability of this study. However, we recruited from two disparate clinical sites in two very different geographic locations to expand the diversity of recruited participants. Fourth, the degree of selection bias among our outpatient methadone clinic cohort is difficult to assess without ascertaining a survey response rate and consequently may impact the reliability of our results. However, we were able to obtain a survey response rate among individuals at our inpatient detoxification site. Those who turned down the survey at the inpatient detoxification site were asleep, were medically unwell, or were preoccupied at the time of survey distribution.

### Future Directions

Future work in this area should include larger patient populations across various sites, which might include non–methadone outpatient substance use clinics. Further, in vivo, randomized controlled studies of promising mental health apps for substance use disorders are needed to establish clinical efficacy. Studies clarifying the effects of socioeconomic status, race, and other factors on digital literacy, smartphone utilization, smartphone ownership, and the acceptability of using apps for substance use interventions among individuals with substance use disorders are needed. Privacy and security concerns around mental health apps will need to be addressed, especially given that individuals with mental health and substance use disorders are particularly vulnerable.

## Abbreviations

A-CHESS: Addiction-Comprehensive Health Enhancement Support System
BWFH: Brigham and Women’s Faulkner Hospital
mHealth: mobile health
MMT: methadone maintenance treatment
PRISMA: Preferred Reporting Items for Systematic Reviews and Meta-Analyses

## Data Availability

All data are represented in the tables and figures displayed in this publication. The data sets that were generated and/or analyzed during this study are available from the corresponding author on reasonable request.
